# Mechanical Stretch Induces Smooth Muscle Cell Dysfunction by Regulating ACE2 via P38/ATF3 and Post-transcriptional Regulation by miR-421

**DOI:** 10.3389/fphys.2020.540591

**Published:** 2021-01-18

**Authors:** Xiaolin Liu, Xinxin Liu, Mengmeng Li, Yu Zhang, Weijia Chen, Meng Zhang, Cheng Zhang, Mei Zhang

**Affiliations:** The Key Laboratory of Cardiovascular Remodeling and Function Research, The State and Shandong Province Joint Key Laboratory of Translational Cardiovascular Medicine, Chinese Ministry of Education, Chinese National Health Commission and Chinese Academy of Medical Sciences, Qilu Hospital of Shandong University, Jinan, China

**Keywords:** angiotensin-converting enzyme 2, mechanical stretch, vascular smooth muscle cells, P38 MAPK, ATF3, miR-421

## Abstract

Mechanical stretch promotes deregulation of vascular smooth muscle cell (VSMC) functions during hypertension-induced vascular remodeling. ACE2 has a wide range of cardiovascular and renal protective effects. Loss of ACE2 is associated with cardiovascular disease, but little is known about the regulation of its expression, especially by abnormal mechanical stretch during hypertension. The present study was designed to investigate the contribution of ACE2 to vascular remodeling under mechanical stretch and to assess the possible underlying mechanisms. The abdominal aortic constriction model was established to mimic the environment *in vivo*. FX-5000T Strain Unit provided mechanical stretch *in vitro*. Overexpression was used to analyze the role of ACE2 played in the proliferation, migration, apoptosis, and collagen metabolism of the VSMCs. RT-qPCR, Western blot, luciferase assay, and ChIP assay were used to elucidate the molecular mechanism of ACE2 expression regulated by stretch. We found that mechanical stretch modulated the expression of the ACE2/Ang-(1–7) and ACE/AngII axis. ACE2 was mechanically sensitive and was involved in the stretch-induced dysfunction of VSMCs. The p38 MAPK/ATF3 pathway and miR-421 participated in the regulation of ACE2. Thus, ACE2 may contribute to the development of vascular remodeling under conditions of mechanical stretch.

## Graphical Abstract

Mechanical stretch may decrease the level of the protective factor ACE2 via the p38 MAPK/ATF3 pathway by prompting ATF3 translocation into the nucleus, which could directly bind to the ACE2 promoter via post-transcriptional regulation by miR-421.

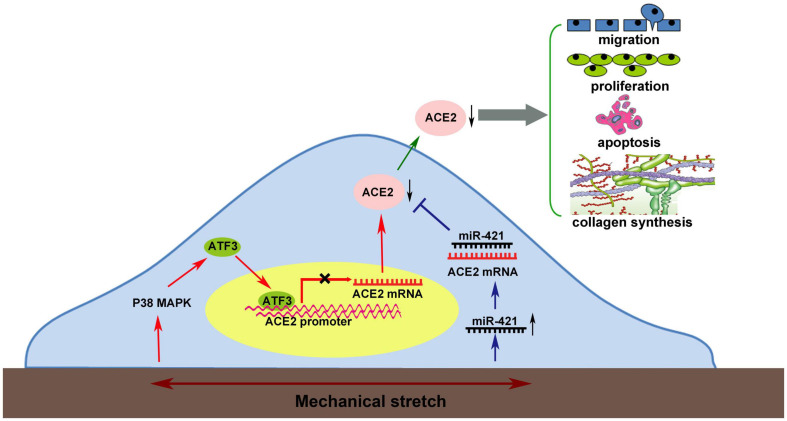

## Introduction

Hypertension is a major risk factor for cardiovascular disease (CVD) and can cause vascular structural and functional abnormalities. During this process, vascular remodeling is a characteristic pathological feature of hypertensive vascular disease (Shi et al., 2018). Hypertension is often accompanied by an increase in the mechanical stretch of the blood vessel wall. As a consequence, excess mechanical stretch affects vascular cells, such as endothelial cells (ECs), vascular smooth muscle cells (VSMCs), and adventitial fibroblasts, and causes the dysfunction of these cells (Hahn and Schwartz, 2009), thus resulting in abnormal vascular structure and function. While stretch can affect all vascular cells, VSMCs located in the media of vascular wall are the main target cells. Under physiological conditions, VSMCs rarely proliferate and migrate, usually performing contractile phenotype (Hu et al., 2014). However, under abnormal mechanical stretch, VSMCs undergo phenotypic transformation, characterized by increased proliferation, migration, and extracellular matrix synthesis, thus resulting in the thickening and stiffening of the arterial wall (Qi et al., 2016; Wang et al., 2019).

The renin angiotensin system (RAS) is closely related to cardiovascular disease. ACE2 is a homolog of ACE but differs from ACE in substrate specificity (Riviere et al., 2005), which is able to cleave Ang II and produce the vasodilating peptide Ang-(1–7) (Gallagher et al., 2006). A wealth of evidence has been uncovered regarding the involvement of ACE2 in cardiovascular disease, including heart failure (Patel et al., 2016), abdominal aortic aneurysms (Thatcher et al., 2014), and atherosclerosis (Zhang et al., 2010). The atheroprotective actions of ACE2 have been shown in a variety of studies. This enzyme is expressed in both normal and diseased vessels of human (Zulli et al., 2008) and animals including rat (Zhang et al., 2019) and mouse (Sahara et al., 2014). It is abundant in different cell types, such as ECs, VSMCs, and macrophages (Zhang et al., 2010, 2019).

It is widely recognized that the RAS system is closely associated with mechanical stimulus (Malhotra et al., 1999; Hu et al., 2014; Abdul-Muneer et al., 2018). Mechanical stretch promoted vascular damage by up-regulating AT1 receptor in SMCs of rats (Hitomi et al., 2006). Others have proved that exposure of endothelial cells to shear stress was reported to decrease the expression of ACE via p53 and the post-transcriptional regulation of miR-143/145 (Kohlstedt et al., 2013). Our team previously demonstrated that ACE could mediate the mechanical stretch-induced phenotype modulation of SMCs (Hu et al., 2014). However, relatively little is known about the mechanisms that regulate ACE2 expression in vascular cells, especially by mechanical stimulus. There was one study finding claiming that ACE2 was strongly abundant in low shear stress-induced carotid plaques (Fraga-Silva et al., 2015), so it is reasonable to speculate that ACE2 is also mechanoresponsive and is possibly involved in mechanical stretch-induced vascular remodeling.

In the current study, the abdominal aortic constriction model was established to investigate the role of ACE2 in vascular modeling *in vivo*. As for *in vitro* studies, VSMCs were exposed to mechanical stretch for the indicated time. The aim of our study is to explore the role of ACE2 played in the functions of VSMCs under mechanical stretch as well as the possible mechanisms that underlie this process. A better understanding of the role that ACE2 plays in the development of vascular remodeling may provide clinicians with opportunities to develop new therapies for treatment.

## Materials and Methods

### Cell Culture and *in vitro* Mechanical Stretch System

Vascular smooth muscle cells of human aorta were purchased from the Sciencell Company (United States) and cultured in smooth muscle cell medium with 5% CO_2_ at 37°C. To apply cyclic mechanical stretch to smooth muscle cells, flexible-bottomed six-well culture plates from Flexcell International Corporation were used. First, the VSMCs were starved for 24 h with serum-free medium. Then, the medium was replaced, and the cells were stimulated with cyclic mechanical stretch. A Flexcell Tension Plus FX-5000T system (Flexcell International Corp., Hillsborough, NC) was used to apply 18% mechanical stretch (pathological) at 1 Hz.

### Abdominal Aortic Constriction of Rats

The abdominal aortic constriction model was performed on rats to induce pressure overload (Cheng et al., 2012). Thirty Wistar rats (male, 200–250 g) were obtained from Beijing University Animal Research Center. The rats were anesthetized with 2% isoflurane. A midline abdominal incision was used to separate the skin, subcutaneous tissue, muscle, and peritoneum. The spleen, stomach, and part of the intestine were pulled to the right of the abdominal cavity and protected with saline gauze. The suprarenal level of the abdominal aorta above the kidneys was tied with 6-0 silk to a polyethylene catheter (PE10), and then the catheter was immediately removed. The sham surgery animals underwent the same procedure without abdominal aortic constriction. The animal experimental protocol conformed to the Guide for the Care and Use of Laboratory Animals published by the US National Institutes of Health and was approved by the ethics committee of Shandong University.

### RNA Extraction and Quantitative Real-Time PCR

TRIzol reagent was used to extract total RNA from the VSMCs. We used spectrophotometry to quantify the concentration of RNA. Then, RNA was reverse-transcribed into cDNA. The SYRB Premix Ex Taq kit (TaKaRa Bio, Japan) was used for real-time PCR. The relative mRNA expression levels of ACE2 and ACE were assessed by the 2^–△△Ct^ method.

### Western Blot Analysis

Protein was extracted from the VSMCs and rat aortas. Total cell lysates were separated with 10% sodium dodecyl sulfate–polyacrylamide gel electrophoresis and transferred to polyvinylidene fluoride membranes. The membranes were incubated with 5% non-fat milk for 2 h and then overnight at 4°C with the corresponding primary antibodies for ACE2 (1:1,000; Abcam, Cambridge, United Kingdom), ACE (1:1,000; Abcam, Cambridge, United Kingdom), activating transcription factor 3 (ATF3; 1:1,000; Abcam, Cambridge, United Kingdom), collagen I (1:1,000; Abcam, Cambridge, United Kingdom), collagen III (1:1,000; Abcam, Cambridge, United Kingdom), total p38 and phosphorylated-p38 (Thr180/Tyr182) (p-p38) (1:1,000; Cell Signaling Technology, MA, United States), total JNK and phosphorylated-JNK (Thr183/Tyr185) (p-JNK) (1:1,000; Cell Signaling Technology, MA, United States), total Erk1/2 and phosphorylated-Erk1/2 (Thr202/Tyr204) (p-Erk1/2) (1:1,000; Cell Signaling Technology, MA, United States), GAPDH (1:1,000; ProteinTech, Wuhan, China), and appropriate secondary antibodies (1:5,000; Abcam, Cambridge, United Kingdom) for 1 h at room temperature. Visualization involved enhanced chemiluminescence plus reagents. Band densities were analyzed by the use of Adobe Photoshop CS6.

### ACE2 Activity Assay

The ACE2 and ACE activity of the VSMCs exposed to cyclic mechanical stretch was determined as described previously (Liu et al., 2011). A reagent, 7-Mca-YVADAPK (Dnp) (R&D Systems, Minneapolis, United States), which is cleaved by ACE2, was used as a fluorogenic substrate. Ten μg total protein extracts were incubated with 1.0 μmol/L 7-Mca-YVADAPK (Dnp) in a final volume of 100 μL reaction buffer at room temperature. EDTA (1 mmol/L) and human ACE2 (25 ng) (R&D Systems, Minneapolis, MN, United States) were designed as negative and positive controls, respectively. Fluorescence kinetics was measured for 4 h by the use of Varioskan Flash (Thermo Fisher Scientific, Worcester, MA, United States) at an excitation wavelength of 320 nm and an emission wavelength of 400 nm. ACE2 activity was defined as the difference in fluorescence with or without the ACE2 inhibitor DX600 (1 μmol/L, Phoenix Pharmaceuticals, Belmont, CA, United States). Data were calculated from triplicate wells and presented as fluorescence unit per hour and normalized to milligram tissue protein.

### ELISA

The VSMCs were subjected to mechanical stretch at the indicated time points and then harvested. The cytoplasmic proteins were extracted using a commercial kit (Pierce), and the protein concentration was determined by bicinchoninic acid assay. The cytoplasmic proteins from each well were stored at −80°C. AngII and Ang-(1–7) levels were evaluated by commercial ELISA kits (AngII-SPI-BIO, France, and Ang 1–7-Bachem, United States, respectively).

### Construction of Adenovirus Vector

To achieve adenovirus-mediated ACE2 overexpression, the adenoviral vector was purchased from GenePharma Co. Ltd. (Shanghai, China), and the full-length coding sequence of human ACE2 C-terminally tagged with green fluorescent protein (GFP) was cloned into the vector. A vector cloned with GFP alone was used as the negative control (NC).

### Bromodeoxyuridine Incorporation Assay

Bromodeoxyuridine (BrdU) incorporation assays were used to determine VSMC proliferation. Cells at passages 4–7 were seeded onto six-well Bioflex plates coated with collagen I and underwent different levels of mechanical stretch. The cells were treated with BrdU labeling medium for 6 h and were fixed with an ethanol fixative at -20°C, incubated at 4°C with anti-BrdU working solution overnight, and then stained with anti-mouse-Ig-fluorescein antibody for 30 min and DAPI for 8 min to label the nuclei. Then, the cells were examined using a microscope.

### Cell Scratch Test

Scratch tests were performed to evaluate the effect of mechanical stretch on VSMC migration. Cells transfected with Ad-ACE2 or Ad-GFP were plated directly onto silicone membranes of Flexcell six-well plates. After reaching confluence, a line of cells was removed with a sterile 100 μL pipette tip across the layer. The culture media were replaced with serum-free medium, and then the VSMCs were exposed to 18% mechanical stretch for 12 h. Then, cells were fixed by incubation with 90% ethanol for 20 min, and the migrated distance was measured along the wound edge using Photoshop CS6 software.

### Transient Transfection

VSMCs were transfected with negative control siRNA or ATF3 siRNA (GenePharma, Shanghai). For miR-421 over-expression and inhibition, miR-421 mimics, and inhibitor (GenePharma, Shanghai) were transfected into the VSMCs. Lipofectamine 3000 was used for transfection.

### Histopathology

The constricted abdominal aortas of rats were dissected and immediately fixed in 4% formalin. The tissue was embedded in paraffin. Successive transverse paraffin sections were cut at a thickness of 5 μm and underwent immunohistochemical incubation with antibodies for ACE2 (1:50) and ACE (1:50) (Abcam, United Kingdom) overnight, followed by incubation with the appropriate secondary antibodies. Signals were amplified with the use of 3,3-diaminobenzidine, counterstained with hematoxylin and analyzed by the use of Image-Pro Plus 6.0 (Media Cybernetics, United States).

### Dual Luciferase Reporter Assay

The 3′-UTR fragments of ACE2 mRNA were cloned into the pmirGLO vector (GenePharma, China). Site-specific mutants were generated using PCR. HEK-293T cells were cultured in DMEM in an atmosphere of 5% CO_2_ at 37°C. Cells at 50–60% confluence were cotransfected with 100 ng of the 3′-UTR luciferase reporter vector and 50 pmol miR-421 mimics (GenePharma, China) using Lipofectamine 3000 transfection reagent (Invitrogen, United States) following the manufacturer’s instructions. After 48 h, firefly and Renilla luciferase activities were detected on a microplate reader (Biotek, United States) using the Dual-Luciferase Reporter Assay System (Promega, United States). To determine the luciferase activity, the ratio of firefly luciferase to Renilla luciferase was calculated for each well.

A series of DNA fragments upstream of the transcription initiation site in the ACE2 promoter [P (-2,000 to -1 bp), P0 (-1,510 to -1 bp), P1 (-227 to 1 bp), P2 (-441 to -218 bp), P3 (-655 to -432 bp), P4 (-868 to -646 bp), P5 (-1,082 to -859 bp), P6 (-1,296 to -1,073 bp) and P7 (-1,510 to -1,289 bp)] were constructed using the pGL3.10-Basic vector. The constructs were transfected into HEK-293T cells with Lipofectamine 3000 transfection reagent (Invitrogen, United States), and the Renilla vector (Promega) was cotransfected to normalize the transfection efficiency, with or without transfection of pcDNA 3.1(+)/ATF3 plasmid. After 48 h, the firefly and Renilla luciferase activities were detected on a microplate reader (Biotek, United States) using the Dual-Luciferase Reporter Assay System (Promega, United States). To determine the luciferase activity, the ratio of firefly luciferase to Renilla luciferase was calculated for each well.

### Chromatin Immunoprecipitation Assay

ChIP was performed using VSMCs (density, 1 × 10^6^ cells). The ChIP assay was performed using the SimpleChIP^®^ Enzymatic Chromatin IP Kit (Magnetic Beads) (CST, United States). The chromatin solution was immunoprecipitated using 5 μg anti-ATF3 (CST, United States) antibody or normal anti-IgG antibody, followed by overnight incubation with magnetic beads at 4°C. Next, the beads were washed multiple times, and the antibody–protein–DNA complexes were eluted. Protein and RNA were removed by treatment with proteinase K and RNase, respectively. Next, PCR was performed using the immunoprecipitated genomic DNA and primers specific for the ATF3 binding site upstream of the transcription start site in the ACE2 promoter. The PCR products obtained were electrophoresed on 1% agarose gel.

### Flow Cytometry

We used the Annexin V-FITC Apoptosis Detection Kit (BIPEC, United States) to determine the apoptotic cells. In brief, cells with or without stretch treatment were resuspended with 400 mL binding buffer at 10^6^ cells/mL, underwent 15 min annexin V-FITC labeling, then 5 min PI labeling, and then were analyzed by flow cytometry in 1 h. In total, 10,000 cells were counted in each assay.

### Statistical Analysis

Each experiment was performed for at least three times, and all data were presented as mean ± SEM. Student’s *t*-test was used to analyze two group comparisons. A comparison among multiple groups was conducted by one-way ANOVA. All statistical tests were calculated using GraphPad Prism 5.00 (GraphPad Software). In this study, a *P*-value less than 0.05 was considered to be statistically significant.

## Results

### *In vivo* Abdominal Aortic Constriction in Rats Influences the Expression of ACE2 and ACE

**FIGURE 1:**
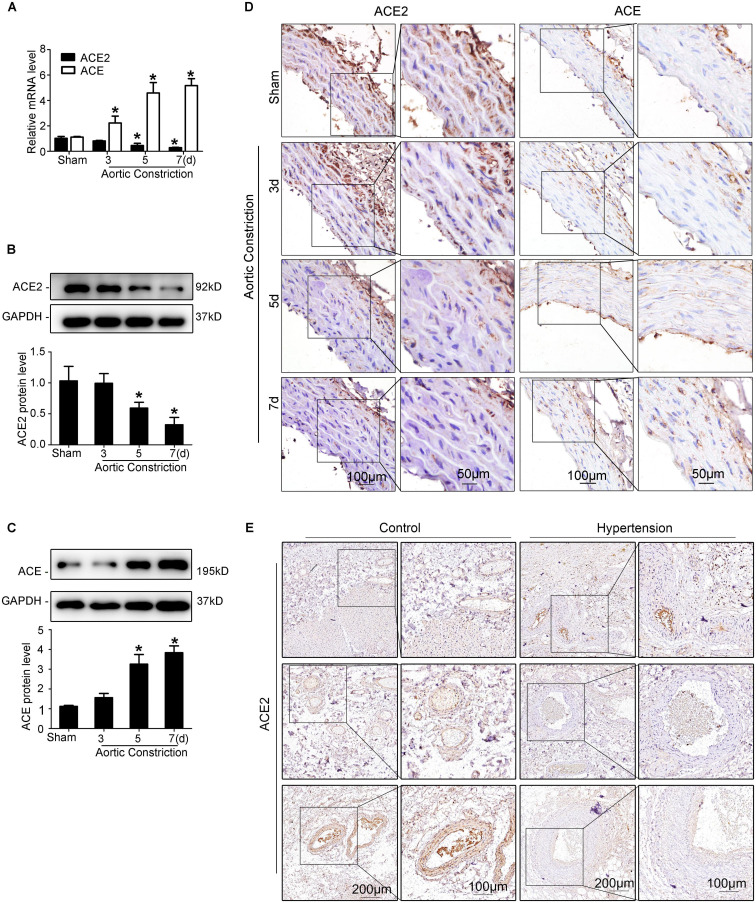
Elevated mechanical stretch suppresses ACE2 and increases ACE expression in the aorta *in vivo*. **(A)** ACE2 and ACE mRNA expression in the aortas of the aortic constriction group and the sham group by RT-qPCR. **(B,C)** ACE2 and ACE protein levels in the aortas of the aortic constriction group and the sham group by Western blot. **(D)** Representative immunohistochemical staining of ACE2 and ACE in sections from the aortas of the aortic constriction group and the sham operation group. **(E)** Representative photomicrographs of immunohistochemical staining of ACE2 in the paracancerous tissues of patients with hypertension or without. Values are expressed as means ± SEM. *P < 0.05 vs. sham (*n* = 4).

The abdominal aortic constriction model was performed in rats to induce pressure overload, which could result in elevated mechanical stretch. We found that the levels of ACE2 time-dependently decreased in the abdominal aorta after aortic constriction by RT-qPCR (Figure 1A), Western blot (Figure 1B), and immunohistochemical staining (Figure 1D) analyses. In contrast, the expression of ACE was up-regulated in the aorta after abdominal aortic constriction compared to the sham group (*P* < 0.05; Figures 1A,C,D). These results showed that both ACE2 and ACE are responsive to mechanical stretch induced by blood pressure. We collected paracancerous tissues from patients with colorectal cancer combining hypertension or not. A total of 12 patients with colorectal cancer were collected, including six patients with a history of hypertension. Representative photomicrographs of immunohistochemical staining showed significant thickening of the arterial wall in patients with hypertension. Our results showed that the expression of ACE2 in smooth muscle cells significantly decreased in those patients with hypertension, including different sizes of arteries (Figure 1E).

### Mechanical Stretch Modulates ACE2/Ang-(1–7) and ACE/AngII Expression in VSMCs *in vitro*

**FIGURE 2:**
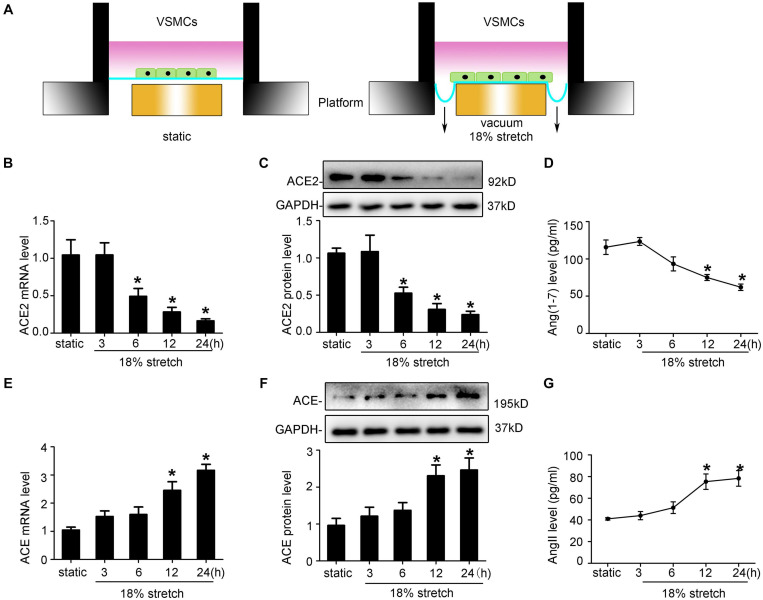
Mechanical stretch modulates ACE2, ACE, Ang-(1–7), and Ang II expression in vascular smooth muscle cells (VSMCs) *in vitro*. **(A)** Schematic diagrams of the mechanical stretch loading system in the current study. VSMCs were subjected to 18% 1 Hz mechanical stretch for the indicated time. **(B,E)** RT-qPCR analysis of ACE2 and ACE mRNA in VSMCs under mechanical stretch for the indicated time. **(C,F)** Western blot analysis of ACE2 and ACE protein expression in VSMCs under mechanical stretch for the indicated time. **(D,G)** Levels of AngII and Ang-(1-7) in VSMCs after exposure to mechanical stretch were assayed by ELISA. Values are expressed as means ± SEM. *P < 0.05 vs. static (*n* = 4).

ACE2 is widely expressed in the human aorta, including intima and media. Thus, endothelial cells may also play an important role in stretch-induced vascular remodeling. As VSMCs are the main target cells of elevated mechanical stretch, we focused on the functions of VSMCs in the present study. Here, using a mechanical stretch loading system (Figure 2A), we found that VSMCs subjected to mechanical stretch for the indicated time showed decreased ACE2 expression at both mRNA and protein levels, with a significant decrease from 6 h (*P* < 0.05; Figures 2B,C), while the expression of ACE was increased (*P* < 0.05; Figures 2E,F). ACE2 enzyme activity also declined under stretch (*P* < 0.05; Supplementary Figure 1). The protective effects of ACE2 were related to its ability to degrade a vasoconstrictor (AngII) and produce a vasodilator [Ang-(1–7)], so we also assessed the levels of Ang-(l–7) and AngII of cytoplasmic protein. The levels of Ang-(l–7) decreased, while the levels of AngII time-dependently increased under stretch (*P* < 0.05; Figures 2D,G).

ACE2 is widely expressed in the human aorta, including intima and media. Thus, endothelial cells may also play an important role in stretch-induced vascular remodeling. As VSMCs are the main target cells of elevated mechanical stretch, we focused on the functions of VSMCs in the present study. By using FX-5000T Strain Unit, which provided mechanical stretch (Figure 2A), we found that VSMCs exposed to mechanical stretch for the indicated time showed a decreased ACE2 expression at both mRNA and protein levels, with a significant decrease from 12 h (*P* < 0.05; Figures 2B,C), while the expression of ACE was increased (*P* < 0.05; Figures 2E,F). ACE2 enzyme activity also declined under stretch (*P* < 0.05; Supplementary Figure 1). The protective effects of ACE2 were related to its ability to degrade a vasoconstrictor (AngII) and produce a vasodilator [Ang-(1–7)] (Thatcher et al., 2014), so we also assessed the levels of Ang-(l–7) and AngII of cytoplasmic protein. The levels of Ang-(l–7) decreased, while the levels of AngII time-dependently increased under stretch (*P* < 0.05; Figures 2D,G).

Considering smaller arteries and arterioles (e.g., mesenteric, kidney arteries) that play a quite important role in regulating systemic blood pressure, we purchased smooth muscle cells isolated from superior mesenteric artery (SMA) and then compared them with the aortic smooth muscle cells that we used before. Our results showed that there was no difference in morphology between the two kinds of cells, nor was there any difference in α-SMA expression. Furthermore, after applying FX-5000T Strain Unit, we found that both types of SMCs exposed to mechanical stretch for the indicated time showed a decreased ACE2 expression (Supplementary Figure 7).

### The Effect of ACE2 on Mechanical Stretch-Induced VSMC Proliferation, Migration, Apoptosis, and Collagen Metabolism

**FIGURE 3:**
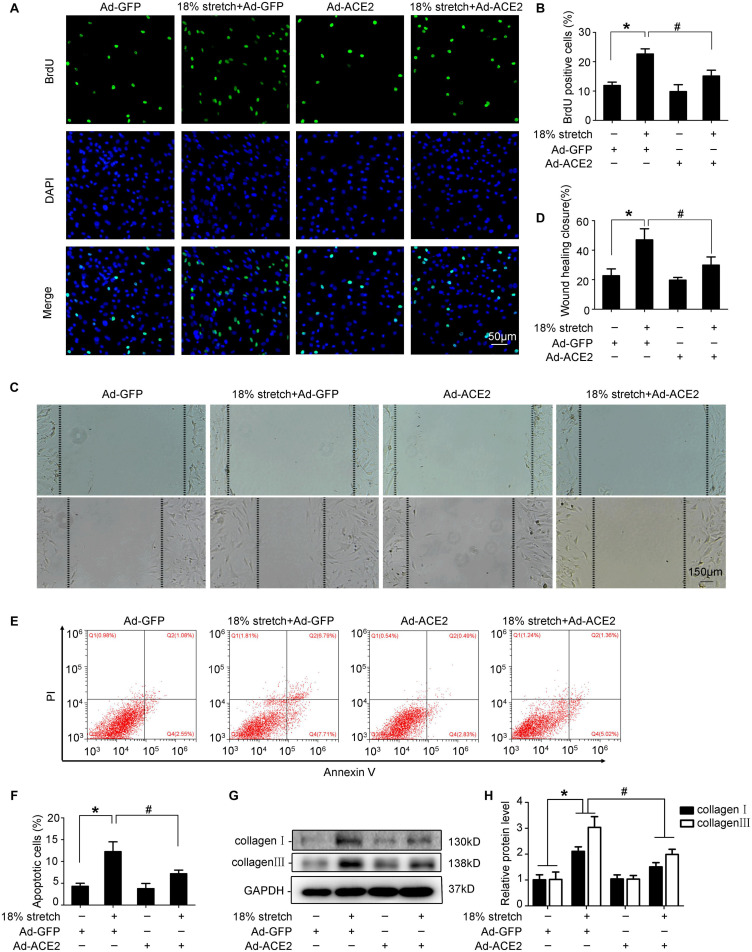
ACE2 was involved in mechanical stretch-induced vascular smooth muscle cells (VSMC) dysfunction. VSMCs were transfected with Ad-ACE2 or Ad-GFP for 24 h and then exposed to mechanical stretch (18% elongation, 1 Hz) for 12 h or not. **(A,B)** Representative views of BrdU-positive cells in different conditions and the percentage of BrdU-positive cells out of total cells. **(C,D)** Representative views of migration distance in different conditions. **(E,F)** Summarized data showing cell apoptosis as determined by flow cytometry analysis in VSMCs. **(G,H)** Western blot analysis of collagen I and collagen III in VSMCs with or without overexpression of ACE2. Values are expressed as means ± SEM. ^∗^*P* < 0.05 vs. Ad-GFP; ^#^*P* < 0.05 vs. 18% stretch + Ad-GFP (*n* = 4).

Mechanical stretch may modulate the functions of VSMCs, such as process of proliferation, migration, and phenotypic transformation; 18% mechanical stretch for 12 h markedly promoted the proliferation of VSMCs compared to static control, and overexpression of ACE2 partly rescued the proliferation increased by stretch (*P* < 0.05; Figures 3A,B). The efficiency of ACE2 overexpression is shown in Supplementary Figure 2. A cell scratch test indicated that the migration distance of VSMCs was obviously stimulated under conditions of stretch, and the promotive effect of mechanical stretch on migration was partly abolished by ACE2 overexpression (*P* < 0.01; Figures 3C,D). Moreover, the overexpression of ACE2 reversed the apoptosis and collagen synthesis induced by mechanical stretch (*P* < 0.05; Figures 3E–H).

Taken together, our results revealed that ACE2 was involved in mechanical stretch-induced VSMC dysfunction.

### The p38 MAPK/ATF3 Pathway Participates in the Expression of ACE2 Under Mechanical Stretch

**FIGURE 4:**
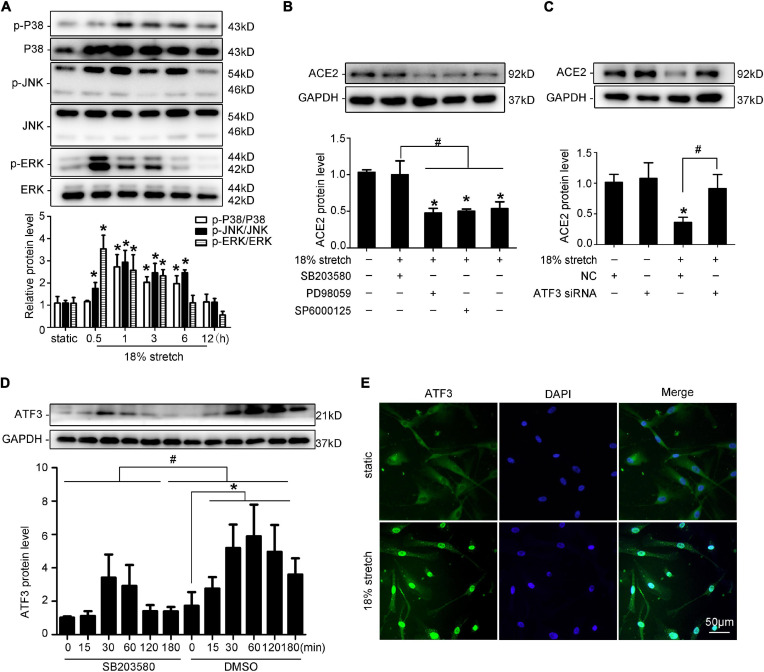
p38 MAPK/ATF3 is involved in ACE2 expression in vascular smooth muscle cells (VSMCs) under 18% mechanical stretch. **(A)** Western blot analysis of the phosphorylation of MAPKs under 18% mechanical stretch for 0–12 h. *P < 0.05 vs. static group. **(B)** Western blot analysis of ACE2 in VSMCs under mechanical stretch pretreated with SB203580, SP600125, or PD98059. *P < 0.05 vs. static control. ^#^*P* < 0.05 vs. 18% stretch control. **(C)** Western blot analysis of ACE2 in VSMCs under 18% stretch or not after transfecting with negative control or ATF3 siRNA. *P < 0.05 vs. NC group, ^#^*P* < 0.05 vs. 18% stretch with NC group. **(D)** Western blot analysis of ATF3 in VSMCs under 18% mechanical stretch with or without pretreatment of SB203580. **(E)** Immunofluorescence using anti-ATF3 was performed under stretch or not.

Previous studies found that mechanical stretch caused the sustained activation of MAPK family members in VSMCs (Haga et al., 2007). A recent study demonstrated that Ang II downregulates ACE2 via the AT1-ERK/p38 MAP kinase pathway (Koka et al., 2008). We found that mechanical stretch induced the phosphorylation of p38 MAPK, JNK, and ERK1/2 (*P* < 0.05, Figure 4A). To validate these results, VSMCs were pretreated with the p38 MAPK inhibitor SB203580, JNK inhibitor SP600125, or ERK1/2 inhibitor PD98059 for 1 h, and then the cells underwent 18% stretch for 12 h. The result showed that SB203580 significantly attenuated the stretch-induced downregulation of ACE2 (*P* < 0.05, Figure 4B). When exposed to 18% mechanical stretch, the expression of ATF3 was increased, and SB203580 blocked the induction (*P* < 0.05, Figure 4D). This result was consistent with previous research claiming that the activation of ATF3 was via the p38 MAPK pathway (Song et al., 2016). Then, we transfected VSMCs with ATF3 siRNA and found that the knockdown of ATF3 reversed the ACE2 level downregulated by mechanical stretch (*P* < 0.05, Figure 4C). The efficiency of silence of ATF3 is shown in Supplementary Figure 3. Immunofluorescence revealed an increased level of ATF3 in the nuclear region under 18% stretch, which indicated that mechanical stretch modulated the expression of ACE2 by promoting the translocation of ATF3 into the nucleus (Figure 4E). Thus, we have full reason to conclude that mechanical stretch may modulate the expression via the p38 MAPK/ATF3 pathway.

### ACE2 Is a Direct Transcriptional Target of ATF3

**FIGURE 5:**
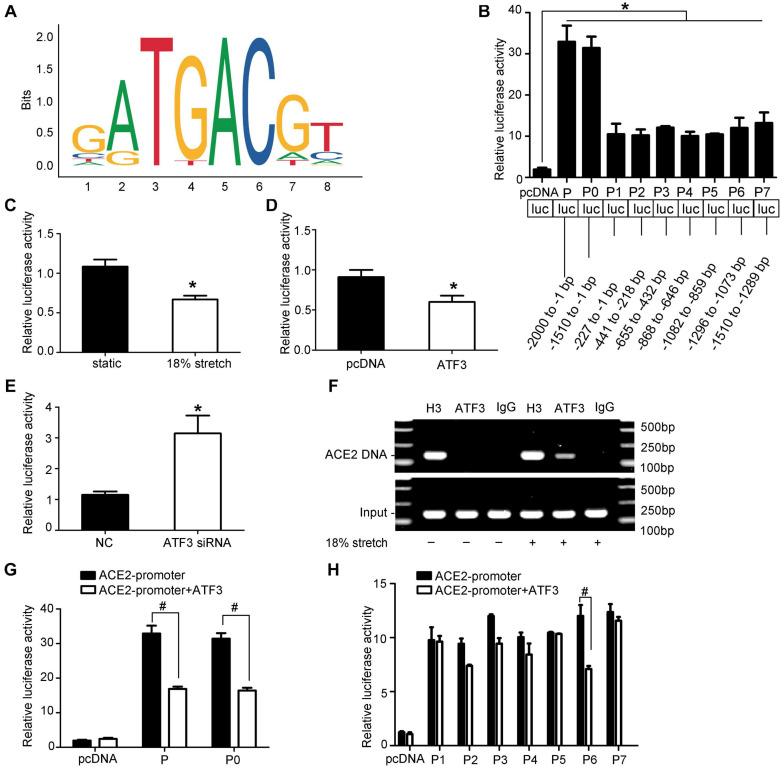
ACE2 is a direct transcriptional target of ATF3. **(A)** The sequence logo of a potential ATF3 binding site in JASPAR. **(B)** The relative luciferase activities of the full ACE2 promoter and the truncated constructs in HEK-293T cells. *P < 0.05 vs. pGL3.10-Basic vector. **(C)** The relative luciferase activities of the full ACE2 promoter P in vascular smooth muscle cells under mechanical stretch for 6 h or not. *P < 0.05 vs. static control. **(D,E)** The relative luciferase activities of the full ACE2 promoter P co-transfected with pcDNA-ATF3 (or ATF3 siRNA). *P < 0.05 vs. control. **(F)** ChIP assay showing ATF3 directly bound to the ACE2 promoter (P6, at the -1,296 to -1,073 bp region). IgG, negative control; H3, positive control; ATF3, ATF3 antibody. **(G,H)** Luciferase activity analysis of P-P7 promoter with or without co-transfection of ATF3. ^#^*P* < 0.05 vs. the corresponding promoter without co-transfection of ATF3 (*n* = 4).

As the results above have shown, the expression of ACE2 may be regulated by stretch via modulating the expression and the location of ATF3. To further identify whether ATF3 could regulate ACE2, we used the JASPAR database (jaspar.genereg.net) to analyze the potential ATF3 binding site in the ACE2 promoter sequence (Figure 5A). Different lengths of the ACE2 promoter, named P-P7, were cloned and inserted into the pGL3.10-Basic vector. Then, these derivatives were, respectively, transfected in HEK-293T cells. The relative luciferase activity of P and P0 were almost 30-fold higher than that of the pGL3.10-Basic vector, and those of P1 to P7 were approximately 10-fold higher (*P* < 0.05, Figure 5B). An analysis of the luciferase activity indicated that these ACE2 promoter fragments were transcriptionally active. Full-length promoter P and Renilla were co-transfected in VSMCs, and then the VSMCs were exposed to stretch for 6 h. The dual luciferase reporter assay showed that stretch significantly suppressed the activity of ACE2 promoter (*P* < 0.05, Figure 5C), which further illustrated that ACE2 is a mechanically sensitive gene. We then co-transfected P and pcDNA-ATF3 (or ATF3 siRNA) in HEK-293T cells and found that overexpression of ATF3 decreased the transcriptional activity of ACE2 promoter (*P* < 0.05, Figure 5D), while ATF3 siRNA significantly enhanced the activity (*P* < 0.05, Figure 5E), which suggested that ATF3 supressed the expression of ACE2. Next, we intended to find the binding site of ATF3 in the promoter of ACE2. Cotransfection with pcDNA–ATF3 significantly decreased the transcriptional activities of the P and P0 derivatives of the ACE2 promoter (*P* < 0.05, Figure 5G), which indicated that the binding site of ATF3 in the promoter of ACE2 was in the region from -1,510 to -1 bp. We then constructed seven fragments of this region (truncated promoter P0) and then cotransfected these fragments with pcDNA-ATF3 into HEK-293T cells. The luciferase activity of P6 significantly decreased (*P* < 0.01, Figure 5H), suggesting that -1,296 to -1,073 bp of the ACE2 promoter may contain the binding site of ATF3. ChIP assays were used to verify whether ATF3 interacted with the -1,296- to -1,073-bp region of the ACE2 promoter. The VSMCs were formaldehyde-crosslinked, and chromatin was prepared and digested to fragments (Supplementary Figure 4). Chromatin was immunoprecipitated using anti-ATF3 antibody or normal anti-IgG antibody. The results of the ChIP assay further verified that ATF3 binds to this transcriptional area of the ACE2 gene (Figure 5F). Taken together, these data suggest that ACE2 is a direct transcriptional target of ATF3.

### miR-421 Is Involved in the Expression of ACE2 Under Mechanical Stretch

**FIGURE 6:**
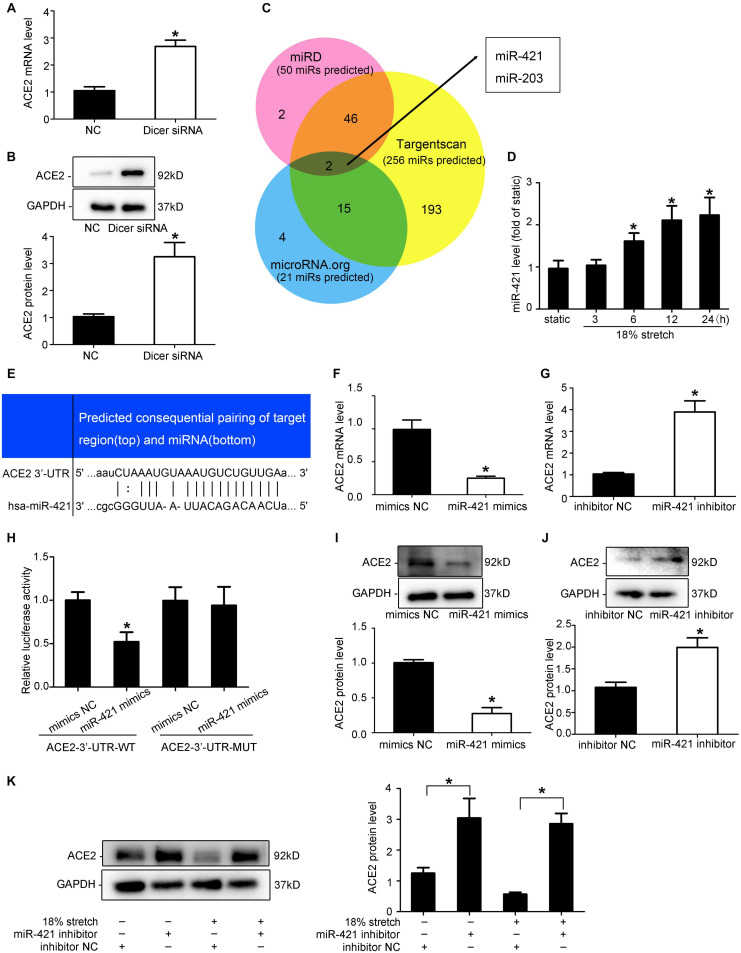
miR-421 is involved in the expression of ACE2 under mechanical stretch. **(A,B)** RT-qPCR and Western blot analysis of ACE2 mRNA and protein expression in vascular smooth muscle cells (VSMCs) transfected with Dicer siRNA. **(C)** Venn diagram of the number of transcription factors in three libraries. **(D)** RT-qPCR analysis of the levels of miR-421 under 18% mechanical stretch for the indicated time. **(E)** Sequence alignment between miR-421 and putative binding sites in the 3′-UTR of ACE2 mRNA. **(H)** Effects of the miR-421 mimics on the activities of the luciferase reporter plasmids with the full-length (Wild) and mutated (Mut) ACE2-3′-UTR. HEK-293T cells were transfected with the ACE2-3′-UTR-WT and ACE2-3′-UTR-mut, together with miR-421 mimics, for 48 h. **(F,G,I,J)** RT-qPCR and Western blot analysis of ACE2 mRNA and protein expression in VSMCs transfected with miR-421 mimics (or inhibitor). **(K)** Western blot analysis of ACE2 protein expression in VSMCs under stretch for 12 h transfected with miR-421 inhibitor. Values are expressed as means ± SEM. *P < 0.05 vs. NC group (*n* = 4).

Kassiri et al. (2009) found that the ACE2 mRNA expression and protein levels did not match in rat myocardial infarction, suggesting that there may be post-transcriptional regulation. To verify whether miRNAs are involved in the regulation of ACE2 expression, Dicer siRNA was used to interfere with the expression of the Dicer enzyme to block the synthesis of miRNAs. RT-qPCR and Western blot results indicated that ACE2 expression was increased after Dicer interference compared with the control group (*P* < 0.05; Figures 6A,B). We used prediction software (TargetScan, miRD, and microRNA.org) to search for putative miRNAs. The three libraries predicted 256, 50, and 21 microRNAs, respectively, and two microRNAs (miR-421 and miR-203) were predicted among all three libraries (Figure 6C). In our research, the levels of miR-421 increased under 18% mechanical stretch (*P* < 0.05, Figure 6D), while the levels of miR-203 decreased (Supplementary Figure 5). Thus, we focused on miR-421 in the following experiments, which were harbored in the binding sites of ACE2 3′-UTR (Figure 6E).

To verify whether miR-421 binds to ACE2-3′-UTR, miR-421 mimics and luciferase reporters containing wild-type (WT) or mutant (Mut) sequences of ACE2-3′-UTR were transfected into HEK-293T cells. Compared with the control group, the miR-421 mimics significantly reduced the ACE2-3′-UTR-WT luciferase activity but had no effect on the ACE2-3′-UTR-Mut luciferase activity (*P* > 0.05, Figure 6H), indicating that miR-421 directly targets ACE2-3′-UTR. To further validate the effect of miR-421 on the expression of ACE2, VSMCs were transfected with miR-421 mimics, inhibitor, or NC sequence. The efficiency of mimics and inhibitor was detected (Supplementary Figure 6). We found that miR-421 mimics significantly reduced the expression of ACE2 in VSMCs (*P* < 0.05; Figures 6F,I), while the miR-421 inhibitor significantly increased the expression of ACE2 (*P* < 0.05; Figures 6G,J). Taken together, the results above suggested that miR-421 negatively regulated the expression of ACE2 by directly binding to the 3′-UTR. However, whether miR-421 is involved in stretch-regulated ACE2 is still unknown. After the VSMCs were transfected with the miR-421 inhibitor, we assessed the expression of ACE2 under mechanical stretch. The miR-421 inhibitor significantly attenuated the stretch-induced downregulation of ACE2 expression (*P* < 0.01, Figure 6K). These results indicated that miR-421 was involved in the regulation of mechanical stretch on ACE2 expression.

## Discussion

From the present findings and those in the literature, Graphical Abstract summarizes a network of molecular events leading to the dysfunction of VSMCs by mechanical stretch. Mechanical stretch may decrease the level of the protective factor ACE2 via the p38 MAPK/ATF3 pathway by prompting ATF3 translocation into the nucleus, which could directly bind to the ACE2 promoter, and via post-transcriptional regulation by miR-421. Downregulation of ACE2 by mechanical stretch leads to vascular remodeling by being involved in the process of VSMC proliferation, migration, apoptosis, and collagen metabolism.

Hypertension is often accompanied by an increase in mechanical stretch of the vascular wall. It is one of the most common factors that cause cardiovascular remodeling, including cardiac and vascular remodeling. Vascular remodeling, often in the aorta, refers to the changes in the structure and function of the arterial wall, which is accompanied by increased stiffness, loss of compliance, and increased inflammatory response in the vascular wall (Galderisi and de Divitiis, 2008; Alghamri et al., 2013). Elevated mechanical stretch promotes proliferation, migration, and collagen synthesis of vascular cells, which causes thickening and stiffness of the vascular wall (Shah et al., 2013; Jufri et al., 2015). As main target of mechanical stretch, VSMCs play a pivotal role in vascular remodeling.

Accumulating evidence demonstrated that “ACE2 is a tissue-specific negative feedback regulator of activated RAS, and the ACE2/Ang-(1–7)-Mas receptor axis plays an important role in regulating blood pressure and cardiovascular remodeling” (Turner and Hooper, 2002; Yamamoto et al., 2006; Zhong et al., 2010; Pan et al., 2018). ACE2 has been shown to reduce hypertension, myocardial hypertrophy, and fibrosis due to Ang II and heart failure induced by pressure overload (Gurley et al., 2006; Grobe et al., 2007; Mercure et al., 2008). Multiple studies have shown that ACE2 plays important roles in VSMC function exposed to various stimuli, including high glucose (Lavrentyev and Malik, 2009), Ang II, Ang II (Patel et al., 2014), and hypoxia (Zhang et al., 2009). In our study, vascular remodeling in a hypertensive model caused by abdominal aortic constriction is associated with a significantly decreased expression of ACE2. As for *in vitro* experiments, the levels of ACE2/Ang-(1–7) and ACE/Ang II in VSMCs showed different trends under the stimulation of mechanical stretch. Stretch promoted the dysfunction of VSMCs, including proliferation, migration, apoptosis, and collagen synthesis. By carrying out gain-of-function experiments, we found that the overexpression of ACE2 by adenovirus significantly reduced the influence of mechanical stretch on SMCs, which suggests a possible role for ACE2 in stretch-induced vascular remodeling. It may seem contradictory that proliferation and apoptosis were simultaneously induced under mechanical stretch in our study. However, we are not the first to report this phenomenon as Professor Li (Ping et al., 2017) and Professor Yan (Cai et al., 2012) also found the same in their research. Although the reason remains largely unclear, there is one explanation that may help. Professor Li found in their study that “dying apoptotic cells were surrounded by the proliferating cells, forming a “dead cell-inducing apoptosis, apoptotic cell-inducing proliferation” vicious circle, leading to accelerated vascular remodeling and eventually diseases” (Ping et al., 2017).

Although ACE2 is profoundly protective in different diseases, it is still difficult in clinical administration due to its instability and degradability. Therefore, we focused on the underlying mechanisms in the hope of finding a new mediator to increase the expression or activity of ACE2. Many signaling pathways have been indicated to be mechano-responsive, which could be influenced by mechanical stretch, including the PI3K/Akt10, PKC11, NFκB12, Rho family GTPases13, and MAPK pathways (Bao et al., 2019). P38 is one of the main members of the MAPK family participating in various cell activities such as cell proliferation, migration, and apoptosis. In our study, we found that stretch increased the phosphorylation level of p38, and the repression of mechanical stretch on ACE2 expression can be weakened by the inhibition of p38. ATF3 is a member of the ATF/CREB gene family, which is an immediate-early gene. Lv et al. (2011) proved that ATF3 can regulate the proliferation and migration of VSMCs. Recent studies have found that ATF3 plays a key role in hypertensive cardiac remodeling (Li et al., 2017). These studies indicate that ATF3 is a mechanically induced gene. Thus, we proposed that p38 MAPK may link the activation of ATF3 with the decreased expression of ACE2 in VSMCs under mechanical stretch. In our study, we confirmed that mechanical stretch downregulated the expression of ACE2 via the p38 MAPK/ATF3 pathway by promoting the expression and translocation of ATF3 into the nucleus. Using dual-luciferase reporter assay and ChIP assays, we demonstrated a new regulatory mechanism of ACE2 expression in which ATF3 binds directly to the promoter and inhibits its expression. These results strongly indicated the pivotal roles of the p38/ATF3 pathway in mechanical stretch-induced ACE2 in VSMCs.

It is known that modulation of gene expression involves various steps, including transcriptional regulation, post-transcriptional regulation, protein synthesis, and degradation. A large number of studies have demonstrated that mechano-miRNAs may also play a role in the progression of vascular remodeling. For example, Professor Zhou found that endothelium-derived miR-126, in response to shear stress, can be delivered to VSMCs and prevent VSMC turnover (Zhou et al., 2013). Lamin A/C negatively regulated by miR-124-3p modulates the apoptosis of vascular smooth muscle cells during cyclic stretch (Bao et al., 2019). We knocked down the expression of the Dicer enzyme to block the synthesis of miRNAs and verified that miRNAs are involved in the regulation of ACE2 expression. By using the prediction soft-wares (TargetScan, miRD, and microRNA.org), two microRNAs (miR-421 and miR-203) were predicted among all three libraries. In our research, the levels of miR-421 increased under mechanical stretch, while the levels of miR-203 decreased, so we focused on miR-421 in the following experiments. Several studies have revealed that miR-421 is very important for proliferation, apoptosis, and tumorigenesis (Meng et al., 2019; Xiao et al., 2019). However, whether miR-421 can be induced by mechanical stretch and whether it is involved in stretch-regulated ACE2 in SMCs are still unknown. In our research, the levels of miR-421 increased under mechanical stretch. Using dual-luciferase reporter assay, ACE2 was validated as a direct target of miR-421, which was consistent with a previous study (Lambert et al., 2014). By performing both gain-of-function experiments, we confirmed that stretch could regulate ACE2 expression via post-transcriptional pathway by miR-421.

In summary, our data revealed that pathological mechanical stretch suppresses the expression of ACE2 via the p38 MAPK/ATF3 pathway and post-transcriptional regulation by miR-421, contributing to promote VSMC dysfunction and vascular remodeling in the hypertension process. A better understanding of the role that ACE2 plays in the development of vascular remodeling may provide clinicians with opportunities to develop new therapies for treatment.

A few limitations need to be mentioned. First, we did not study the mechanisms of how ACE2 influences the functions of VSMCs. Second, the role of ACE2 would be better supported by its overexpression *in vivo*.

## Data Availability Statement

All datasets generated for this study are included in the article/Supplementary Material.

## Ethics Statement

The study was approved by the Medical Ethics Committee of Qilu Hospital of Shandong University (KYLL-202011-121).

## Author Contributions

XLL, XXL, and ML designed the experiments. XLL, YZ, and WC performed the experiments. MenZ and CZ analyzed the data. XLL and MeiZ wrote the manuscript. All authors approved the final version of the manuscript.

## Conflict of Interest

The authors declare that the research was conducted in the absence of any commercial or financial relationships that could be construed as a potential conflict of interest.
