# Supplementary figures and images for: Mechanical Stretch Induces Smooth Muscle Cell Dysfunction by Regulating ACE2 via P38/ATF3 and Post-transcriptional Regulation by miR-421
Source: Front Physiol. 2021 Jan 18;11:540591. doi: 10.3389/fphys.2020.540591 (PMC7848200; doi:10.3389/fphys.2020.540591)

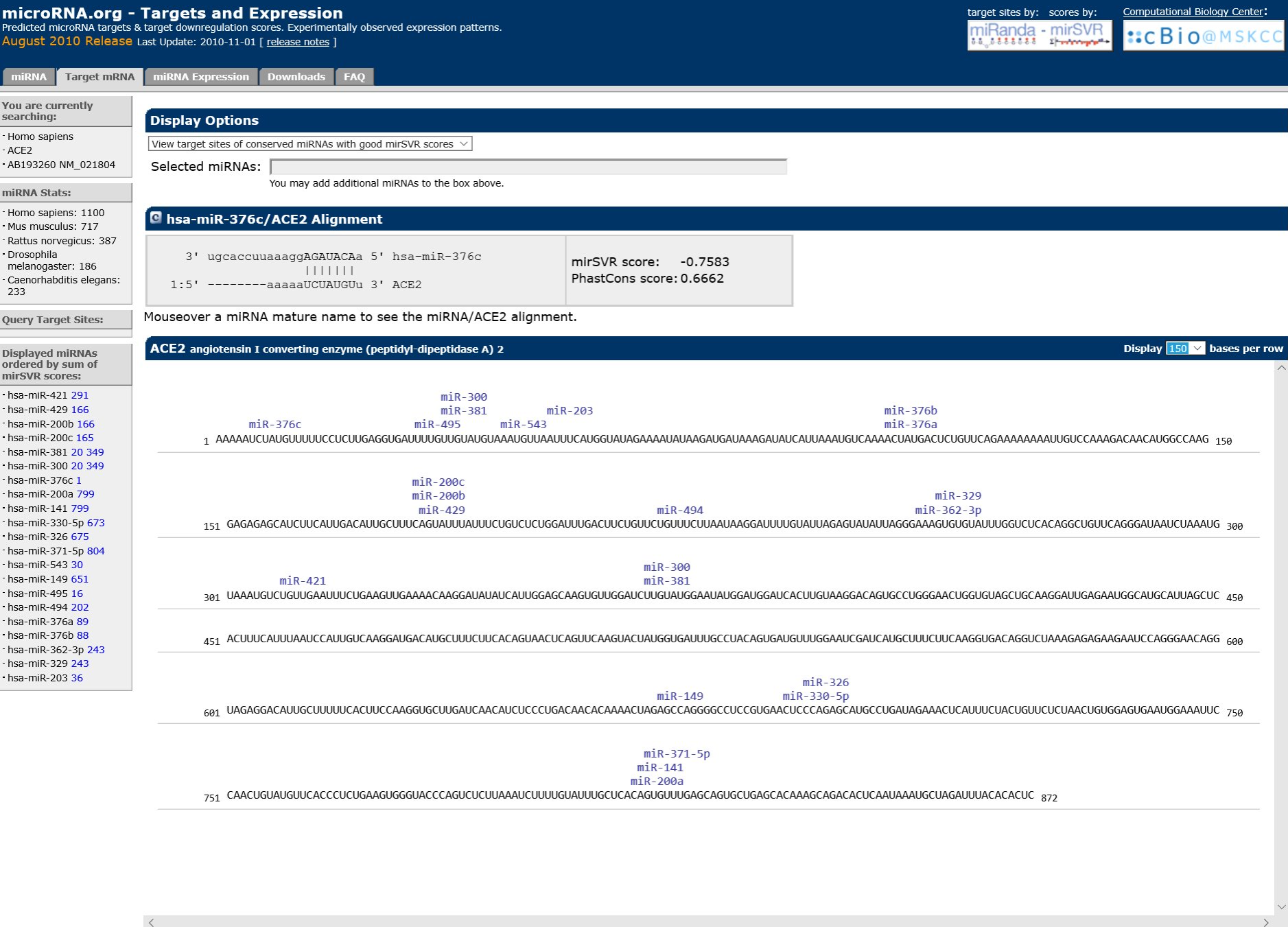

Supplement: Supplementary file 1 [file Table_1.DOCX]
