# Supplementary material for: Mechanical Stretch Induces Smooth Muscle Cell Dysfunction by Regulating ACE2 via P38/ATF3 and Post-transcriptional Regulation by miR-421
Source: Front Physiol. 2021 Jan 18;11:540591. doi: 10.3389/fphys.2020.540591 (PMC7848200; doi:10.3389/fphys.2020.540591)
Supplement: Supplementary file 2 [file Table_2.DOCX]

| [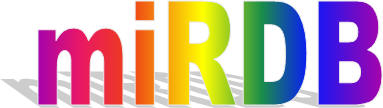](http://mirdb.org/index.html) |
| --- |

**Gene 59272 is predicted to be targeted by 50 miRNAs in miRDB.**

| **Target Detail** | **Target Rank** | **Target Score** | **miRNA Name** | **Gene Symbol** | **Gene Description** |
| --- | --- | --- | --- | --- | --- |
| [Details](http://mirdb.org/cgi-bin/target_detail.cgi?targetID=1480972) | 1 | 84 | [hsa-miR-520a-5p](http://mirdb.org/cgi-bin/mature_mir.cgi?name=hsa-miR-520a-5p) | ACE2 | angiotensin I converting enzyme 2 |
| [Details](http://mirdb.org/cgi-bin/target_detail.cgi?targetID=1972606) | 2 | 84 | [hsa-miR-525-5p](http://mirdb.org/cgi-bin/mature_mir.cgi?name=hsa-miR-525-5p) | ACE2 | angiotensin I converting enzyme 2 |
| [Details](http://mirdb.org/cgi-bin/target_detail.cgi?targetID=1430849) | 3 | 84 | [hsa-miR-3925-5p](http://mirdb.org/cgi-bin/mature_mir.cgi?name=hsa-miR-3925-5p) | ACE2 | angiotensin I converting enzyme 2 |
| [Details](http://mirdb.org/cgi-bin/target_detail.cgi?targetID=1166777) | 4 | 83 | [hsa-miR-4524a-3p](http://mirdb.org/cgi-bin/mature_mir.cgi?name=hsa-miR-4524a-3p) | ACE2 | angiotensin I converting enzyme 2 |
| [Details](http://mirdb.org/cgi-bin/target_detail.cgi?targetID=911038) | 5 | 83 | [hsa-miR-500a-5p](http://mirdb.org/cgi-bin/mature_mir.cgi?name=hsa-miR-500a-5p) | ACE2 | angiotensin I converting enzyme 2 |
| [Details](http://mirdb.org/cgi-bin/target_detail.cgi?targetID=3101308) | 6 | 82 | [hsa-miR-4520-2-3p](http://mirdb.org/cgi-bin/mature_mir.cgi?name=hsa-miR-4520-2-3p) | ACE2 | angiotensin I converting enzyme 2 |
| [Details](http://mirdb.org/cgi-bin/target_detail.cgi?targetID=995263) | 7 | 80 | [hsa-miR-1246](http://mirdb.org/cgi-bin/mature_mir.cgi?name=hsa-miR-1246) | ACE2 | angiotensin I converting enzyme 2 |
| [Details](http://mirdb.org/cgi-bin/target_detail.cgi?targetID=2509887) | 8 | 78 | [hsa-miR-10397-5p](http://mirdb.org/cgi-bin/mature_mir.cgi?name=hsa-miR-10397-5p) | ACE2 | angiotensin I converting enzyme 2 |
| [Details](http://mirdb.org/cgi-bin/target_detail.cgi?targetID=2456742) | 9 | 77 | [hsa-miR-936](http://mirdb.org/cgi-bin/mature_mir.cgi?name=hsa-miR-936) | ACE2 | angiotensin I converting enzyme 2 |
| [Details](http://mirdb.org/cgi-bin/target_detail.cgi?targetID=585066) | 10 | 76 | [hsa-miR-421](http://mirdb.org/cgi-bin/mature_mir.cgi?name=hsa-miR-421) | ACE2 | angiotensin I converting enzyme 2 |
| [Details](http://mirdb.org/cgi-bin/target_detail.cgi?targetID=2256221) | 11 | 74 | [hsa-miR-500b-5p](http://mirdb.org/cgi-bin/mature_mir.cgi?name=hsa-miR-500b-5p) | ACE2 | angiotensin I converting enzyme 2 |
| [Details](http://mirdb.org/cgi-bin/target_detail.cgi?targetID=1332402) | 12 | 74 | [hsa-miR-4693-5p](http://mirdb.org/cgi-bin/mature_mir.cgi?name=hsa-miR-4693-5p) | ACE2 | angiotensin I converting enzyme 2 |
| [Details](http://mirdb.org/cgi-bin/target_detail.cgi?targetID=3249753) | 13 | 74 | [hsa-miR-362-5p](http://mirdb.org/cgi-bin/mature_mir.cgi?name=hsa-miR-362-5p) | ACE2 | angiotensin I converting enzyme 2 |
| [Details](http://mirdb.org/cgi-bin/target_detail.cgi?targetID=632873) | 14 | 72 | [hsa-miR-4441](http://mirdb.org/cgi-bin/mature_mir.cgi?name=hsa-miR-4441) | ACE2 | angiotensin I converting enzyme 2 |
| [Details](http://mirdb.org/cgi-bin/target_detail.cgi?targetID=2944667) | 15 | 71 | [hsa-miR-3646](http://mirdb.org/cgi-bin/mature_mir.cgi?name=hsa-miR-3646) | ACE2 | angiotensin I converting enzyme 2 |
| [Details](http://mirdb.org/cgi-bin/target_detail.cgi?targetID=515234) | 16 | 71 | [hsa-miR-6852-3p](http://mirdb.org/cgi-bin/mature_mir.cgi?name=hsa-miR-6852-3p) | ACE2 | angiotensin I converting enzyme 2 |
| [Details](http://mirdb.org/cgi-bin/target_detail.cgi?targetID=3411419) | 17 | 68 | [hsa-miR-4288](http://mirdb.org/cgi-bin/mature_mir.cgi?name=hsa-miR-4288) | ACE2 | angiotensin I converting enzyme 2 |
| [Details](http://mirdb.org/cgi-bin/target_detail.cgi?targetID=3394378) | 18 | 68 | [hsa-miR-203b-3p](http://mirdb.org/cgi-bin/mature_mir.cgi?name=hsa-miR-203b-3p) | ACE2 | angiotensin I converting enzyme 2 |
| [Details](http://mirdb.org/cgi-bin/target_detail.cgi?targetID=2004911) | 19 | 67 | [hsa-miR-3658](http://mirdb.org/cgi-bin/mature_mir.cgi?name=hsa-miR-3658) | ACE2 | angiotensin I converting enzyme 2 |
| [Details](http://mirdb.org/cgi-bin/target_detail.cgi?targetID=1060149) | 20 | 66 | [hsa-miR-6859-5p](http://mirdb.org/cgi-bin/mature_mir.cgi?name=hsa-miR-6859-5p) | ACE2 | angiotensin I converting enzyme 2 |
| [Details](http://mirdb.org/cgi-bin/target_detail.cgi?targetID=2126305) | 21 | 66 | [hsa-miR-3125](http://mirdb.org/cgi-bin/mature_mir.cgi?name=hsa-miR-3125) | ACE2 | angiotensin I converting enzyme 2 |
| [Details](http://mirdb.org/cgi-bin/target_detail.cgi?targetID=3080570) | 22 | 66 | [hsa-miR-3916](http://mirdb.org/cgi-bin/mature_mir.cgi?name=hsa-miR-3916) | ACE2 | angiotensin I converting enzyme 2 |
| [Details](http://mirdb.org/cgi-bin/target_detail.cgi?targetID=874748) | 23 | 65 | [hsa-miR-3187-3p](http://mirdb.org/cgi-bin/mature_mir.cgi?name=hsa-miR-3187-3p) | ACE2 | angiotensin I converting enzyme 2 |
| [Details](http://mirdb.org/cgi-bin/target_detail.cgi?targetID=2016397) | 24 | 64 | [hsa-miR-4682](http://mirdb.org/cgi-bin/mature_mir.cgi?name=hsa-miR-4682) | ACE2 | angiotensin I converting enzyme 2 |
| [Details](http://mirdb.org/cgi-bin/target_detail.cgi?targetID=122643) | 25 | 64 | [hsa-miR-5680](http://mirdb.org/cgi-bin/mature_mir.cgi?name=hsa-miR-5680) | ACE2 | angiotensin I converting enzyme 2 |
| [Details](http://mirdb.org/cgi-bin/target_detail.cgi?targetID=1215166) | 26 | 64 | [hsa-miR-4270](http://mirdb.org/cgi-bin/mature_mir.cgi?name=hsa-miR-4270) | ACE2 | angiotensin I converting enzyme 2 |
| [Details](http://mirdb.org/cgi-bin/target_detail.cgi?targetID=1881208) | 27 | 62 | [hsa-miR-302c-5p](http://mirdb.org/cgi-bin/mature_mir.cgi?name=hsa-miR-302c-5p) | ACE2 | angiotensin I converting enzyme 2 |
| [Details](http://mirdb.org/cgi-bin/target_detail.cgi?targetID=1736043) | 28 | 62 | [hsa-miR-6873-3p](http://mirdb.org/cgi-bin/mature_mir.cgi?name=hsa-miR-6873-3p) | ACE2 | angiotensin I converting enzyme 2 |
| [Details](http://mirdb.org/cgi-bin/target_detail.cgi?targetID=72862) | 29 | 61 | [hsa-miR-4477a](http://mirdb.org/cgi-bin/mature_mir.cgi?name=hsa-miR-4477a) | ACE2 | angiotensin I converting enzyme 2 |
| [Details](http://mirdb.org/cgi-bin/target_detail.cgi?targetID=2399441) | 30 | 61 | [hsa-miR-1305](http://mirdb.org/cgi-bin/mature_mir.cgi?name=hsa-miR-1305) | ACE2 | angiotensin I converting enzyme 2 |
| [Details](http://mirdb.org/cgi-bin/target_detail.cgi?targetID=2359346) | 31 | 61 | [hsa-miR-3909](http://mirdb.org/cgi-bin/mature_mir.cgi?name=hsa-miR-3909) | ACE2 | angiotensin I converting enzyme 2 |
| [Details](http://mirdb.org/cgi-bin/target_detail.cgi?targetID=1291232) | 32 | 61 | [hsa-miR-7850-5p](http://mirdb.org/cgi-bin/mature_mir.cgi?name=hsa-miR-7850-5p) | ACE2 | angiotensin I converting enzyme 2 |
| [Details](http://mirdb.org/cgi-bin/target_detail.cgi?targetID=570102) | 33 | 60 | [hsa-miR-6755-3p](http://mirdb.org/cgi-bin/mature_mir.cgi?name=hsa-miR-6755-3p) | ACE2 | angiotensin I converting enzyme 2 |
| [Details](http://mirdb.org/cgi-bin/target_detail.cgi?targetID=2095456) | 34 | 60 | [hsa-miR-6754-5p](http://mirdb.org/cgi-bin/mature_mir.cgi?name=hsa-miR-6754-5p) | ACE2 | angiotensin I converting enzyme 2 |
| [Details](http://mirdb.org/cgi-bin/target_detail.cgi?targetID=2748068) | 35 | 60 | [hsa-miR-3908](http://mirdb.org/cgi-bin/mature_mir.cgi?name=hsa-miR-3908) | ACE2 | angiotensin I converting enzyme 2 |
| [Details](http://mirdb.org/cgi-bin/target_detail.cgi?targetID=1779648) | 36 | 60 | [hsa-miR-3529-3p](http://mirdb.org/cgi-bin/mature_mir.cgi?name=hsa-miR-3529-3p) | ACE2 | angiotensin I converting enzyme 2 |
| [Details](http://mirdb.org/cgi-bin/target_detail.cgi?targetID=1588102) | 37 | 60 | [hsa-miR-6804-5p](http://mirdb.org/cgi-bin/mature_mir.cgi?name=hsa-miR-6804-5p) | ACE2 | angiotensin I converting enzyme 2 |
| [Details](http://mirdb.org/cgi-bin/target_detail.cgi?targetID=1447344) | 38 | 58 | [hsa-miR-1303](http://mirdb.org/cgi-bin/mature_mir.cgi?name=hsa-miR-1303) | ACE2 | angiotensin I converting enzyme 2 |
| [Details](http://mirdb.org/cgi-bin/target_detail.cgi?targetID=374950) | 39 | 58 | [hsa-miR-4272](http://mirdb.org/cgi-bin/mature_mir.cgi?name=hsa-miR-4272) | ACE2 | angiotensin I converting enzyme 2 |
| [Details](http://mirdb.org/cgi-bin/target_detail.cgi?targetID=569619) | 40 | 57 | [hsa-miR-6806-5p](http://mirdb.org/cgi-bin/mature_mir.cgi?name=hsa-miR-6806-5p) | ACE2 | angiotensin I converting enzyme 2 |
| [Details](http://mirdb.org/cgi-bin/target_detail.cgi?targetID=1037381) | 41 | 57 | [hsa-miR-6822-3p](http://mirdb.org/cgi-bin/mature_mir.cgi?name=hsa-miR-6822-3p) | ACE2 | angiotensin I converting enzyme 2 |
| [Details](http://mirdb.org/cgi-bin/target_detail.cgi?targetID=1750891) | 42 | 56 | [hsa-miR-212-5p](http://mirdb.org/cgi-bin/mature_mir.cgi?name=hsa-miR-212-5p) | ACE2 | angiotensin I converting enzyme 2 |
| [Details](http://mirdb.org/cgi-bin/target_detail.cgi?targetID=1592595) | 43 | 54 | [hsa-miR-4773](http://mirdb.org/cgi-bin/mature_mir.cgi?name=hsa-miR-4773) | ACE2 | angiotensin I converting enzyme 2 |
| [Details](http://mirdb.org/cgi-bin/target_detail.cgi?targetID=706981) | 44 | 54 | [hsa-miR-4766-5p](http://mirdb.org/cgi-bin/mature_mir.cgi?name=hsa-miR-4766-5p) | ACE2 | angiotensin I converting enzyme 2 |
| [Details](http://mirdb.org/cgi-bin/target_detail.cgi?targetID=2641109) | 45 | 54 | [hsa-miR-4786-5p](http://mirdb.org/cgi-bin/mature_mir.cgi?name=hsa-miR-4786-5p) | ACE2 | angiotensin I converting enzyme 2 |
| [Details](http://mirdb.org/cgi-bin/target_detail.cgi?targetID=2453578) | 46 | 53 | [hsa-miR-767-3p](http://mirdb.org/cgi-bin/mature_mir.cgi?name=hsa-miR-767-3p) | ACE2 | angiotensin I converting enzyme 2 |
| [Details](http://mirdb.org/cgi-bin/target_detail.cgi?targetID=1214217) | 47 | 52 | [hsa-miR-3185](http://mirdb.org/cgi-bin/mature_mir.cgi?name=hsa-miR-3185) | ACE2 | angiotensin I converting enzyme 2 |
| [Details](http://mirdb.org/cgi-bin/target_detail.cgi?targetID=2024741) | 48 | 52 | [hsa-miR-10522-5p](http://mirdb.org/cgi-bin/mature_mir.cgi?name=hsa-miR-10522-5p) | ACE2 | angiotensin I converting enzyme 2 |
| [Details](http://mirdb.org/cgi-bin/target_detail.cgi?targetID=2577999) | 49 | 51 | [hsa-miR-4308](http://mirdb.org/cgi-bin/mature_mir.cgi?name=hsa-miR-4308) | ACE2 | angiotensin I converting enzyme 2 |
| [Details](http://mirdb.org/cgi-bin/target_detail.cgi?targetID=912833) | 50 | 51 | [hsa-miR-4677-3p](http://mirdb.org/cgi-bin/mature_mir.cgi?name=hsa-miR-4677-3p) | ACE2 | angiotensin I converting enzyme 2 |
