# Supplementary material for: Mechanical Stretch Induces Smooth Muscle Cell Dysfunction by Regulating ACE2 via P38/ATF3 and Post-transcriptional Regulation by miR-421
Source: Front Physiol. 2021 Jan 18;11:540591. doi: 10.3389/fphys.2020.540591 (PMC7848200; doi:10.3389/fphys.2020.540591)
Supplement: Supplementary file 4 [file Data_Sheet_1.docx]

Supplementary Material

**Table S1. Primer pairs of target genes used for real time RT-qPCR in this study**

| **Genes** | **Forward** | **Reverse** |
| --- | --- | --- |
| Rat ACE2 | CATTGGAGCA AGTGTTGGAT | GAGCTAATGCATGCCATTCA |
| Rat ACE | GGGCAGTGGCTACGAGCATG | AGTTCTCCTGGTGATGCTTC |
| Rat GAPDH | TGATTCTACCCACGGCAAGTT | TGATGGGTTTCCCATTGATGA |
| Human ACE2 | CATTGGAGCAAGTGTTGGATCTT | GAGCTAATGCATGCCATTCTCA |
| Human ACE | GCGGCTCTTCCAGGAGCTGC | CTGCGCCCACATGTTCCCCA |
| Human GAPDH | GCACCGTCAAGGCTGAGAAC | TGGTGAAGACGCCAGTGGA |
| MiR-421 | CTCACTCACATCAACAGACATTAATT | TATGGTTGTTCTGCTCTCTGTGTC |
| MiR-203 | CGGGTGAAATGTTTAGG | GAGCAGGCTGGAGAA |

**Table S2. Antibodies used in this study**

| **Primary antibodies** | **Host** | **ID code** | **Dilution and supplier** | **Application** |
| --- | --- | --- | --- | --- |
| ACE2 | Rabbit | ab108252 | 1:1000(WB),1:50(IHC) ;Abcam, Cambridge, MA | WB,IHC |
| ACE | Rabbit | ab75762 | 1:1000(WB),1:50(IHC) ;Abcam, Cambridge, MA | WB,IHC |
| GAPDH | Mouse | 60004-1-Ig | 1:1000; ProteinTech Group, ProteinTech, Wuhan, China | WB |
| ATF3 | Rabbit | ab207434 | 1:1000(WB),1:200(IF) ; Abcam, Cambridge, MA | WB,IF |
| ATF3 | Rabbit | [33593](https://www.cst-c.com.cn/products/primary-antibodies/atf-3-d2y5w-rabbit-mab/33593?site-search-type=Products) | 1:50; Cell Signaling, Danvers, MA | ChIP |
| p-P38 | Rabbit | [4511](https://www.cst-c.com.cn/products/primary-antibodies/phospho-p38-mapk-thr180-tyr182-d3f9-xp-rabbit-mab/4511?site-search-type=Products) | 1:1000; Cell Signaling, Danvers, MA | WB |
| P38 | Rabbit | [8690](https://www.cst-c.com.cn/products/primary-antibodies/p38-mapk-d13e1-xp-rabbit-mab/8690?site-search-type=Products) | 1:1000; Cell Signaling, Danvers, MA | WB |
| p-JNK | Rabbit | [4668](https://www.cst-c.com.cn/products/primary-antibodies/phospho-sapk-jnk-thr183-tyr185-81e11-rabbit-mab/4668?site-search-type=Products) | 1:1000; Cell Signaling, Danvers, MA | WB |
| JNK | Rabbit | [9252](https://www.cst-c.com.cn/products/primary-antibodies/sapk-jnk-antibody/9252?site-search-type=Products) | 1:1000; Cell Signaling, Danvers, MA | WB |
| p-Erk1/2 | Rabbit | [4370](https://www.cst-c.com.cn/products/primary-antibodies/phospho-p44-42-mapk-erk1-2-thr202-tyr204-d13-14-4e-xp-rabbit-mab/4370?site-search-type=Products) | 1:1000; Cell Signaling, Danvers, MA | WB |
| Erk1/2 | Rabbit | [4695](https://www.cst-c.com.cn/products/primary-antibodies/p44-42-mapk-erk1-2-137f5-rabbit-mab/4695?site-search-type=Products) | 1:1000; Cell Signaling, Danvers, MA | WB |
| CollagenⅠ | Rabbit | ab34710 | 1:1000(WB);Abcam, Cambridge, MA | WB |
| Collagen Ⅲ | Rabbit | ab7778 | 1:1000(WB) ;Abcam, Cambridge, MA | WB |

**Table S3. The sequences of siRNA, mimics and inhibitor for target genes used in the study**

| ATF3siRNA-1 | 5'-UAUCUGUUGGAUAAAGAGGUU-3'  5'-CCUCUUUAUCCAACAGAUAAA-3' |
| --- | --- |
| ATF3siRNA-2 | 5’-GGCGACGAGAAAGAAAUAATT-3’  5’-UUAUUUCUUUCUCGUCGCCTT-3’ |
| ATF3siRNA-3 | 5'-UUCUCCGAACGUGUCACGUTT-3'  5'-ACGUGACACGUUCGGAGAATT-3' |
| Dicer siRNA | 5’-UUUGUUGCGAGGCUGAUUCTT-3’  5’-GAAUCAGCCUCGCAACAAATT-3’ |
| Negative control siRNA | 5'-UUCUCCGAACGUGUCACGUTT-3'  5'-ACGUGACACGUUCGGAGAATT-3' |
| miR-421mimics | 5’-AUCAACAGACAUUAAUUGGGCGC-3’  5’-GCCCAAUUAAUGUCUGUUGAUUU-3’ |
| miR-421inhibitor | 5’-GCGCCCAAUUAAUGUCUGUUGAU-3’ |
| microRNA inhibitor NC | 5’-CAGUACUUUUGUGUAGUACAA-3’ |


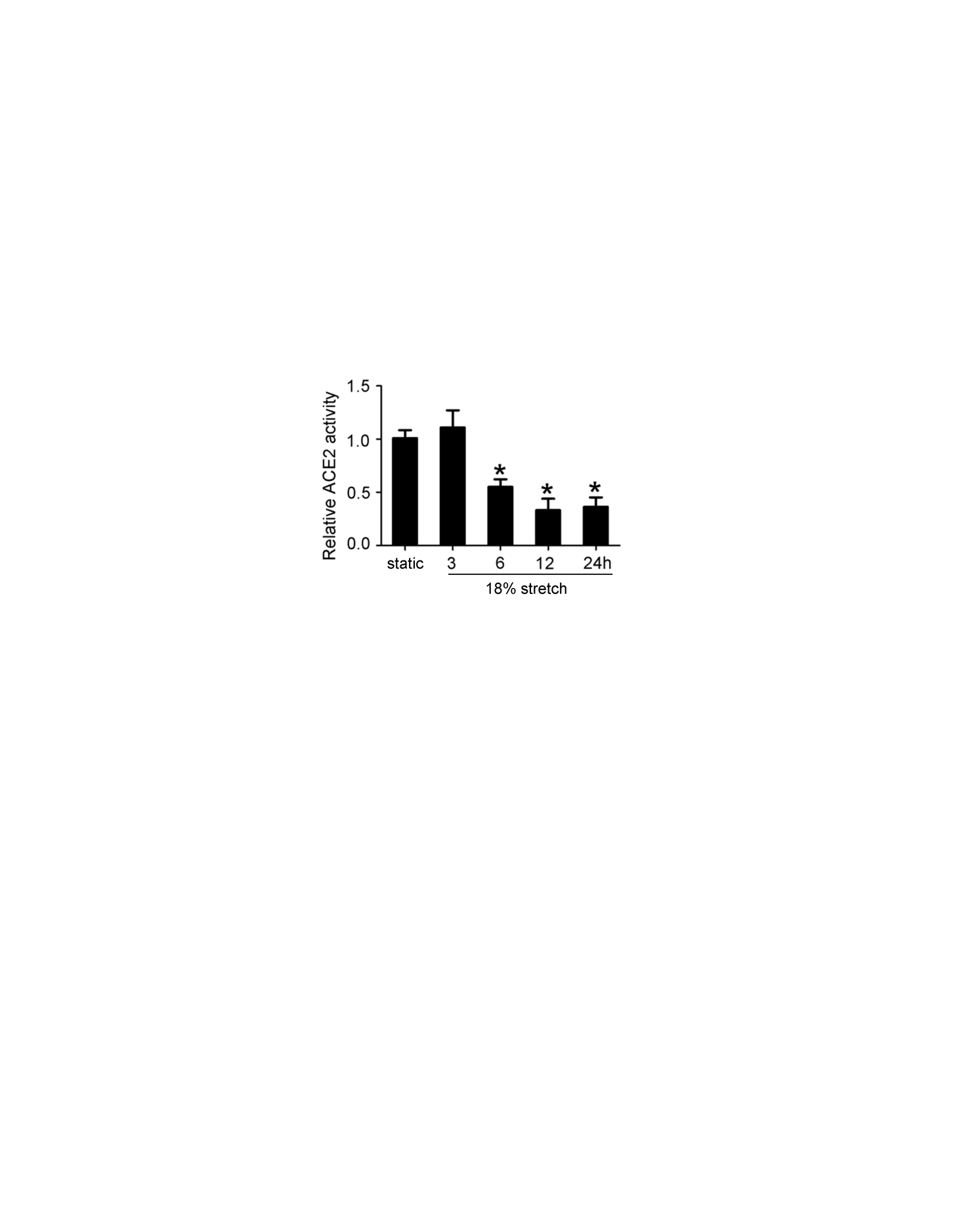


**Supplementary Figure 1.** ACE2 activity in HASMCs was determined under mechanical stretch at different time points. Values are expressed as the means ± SEM. * P<0.05 versus static group (n=4).


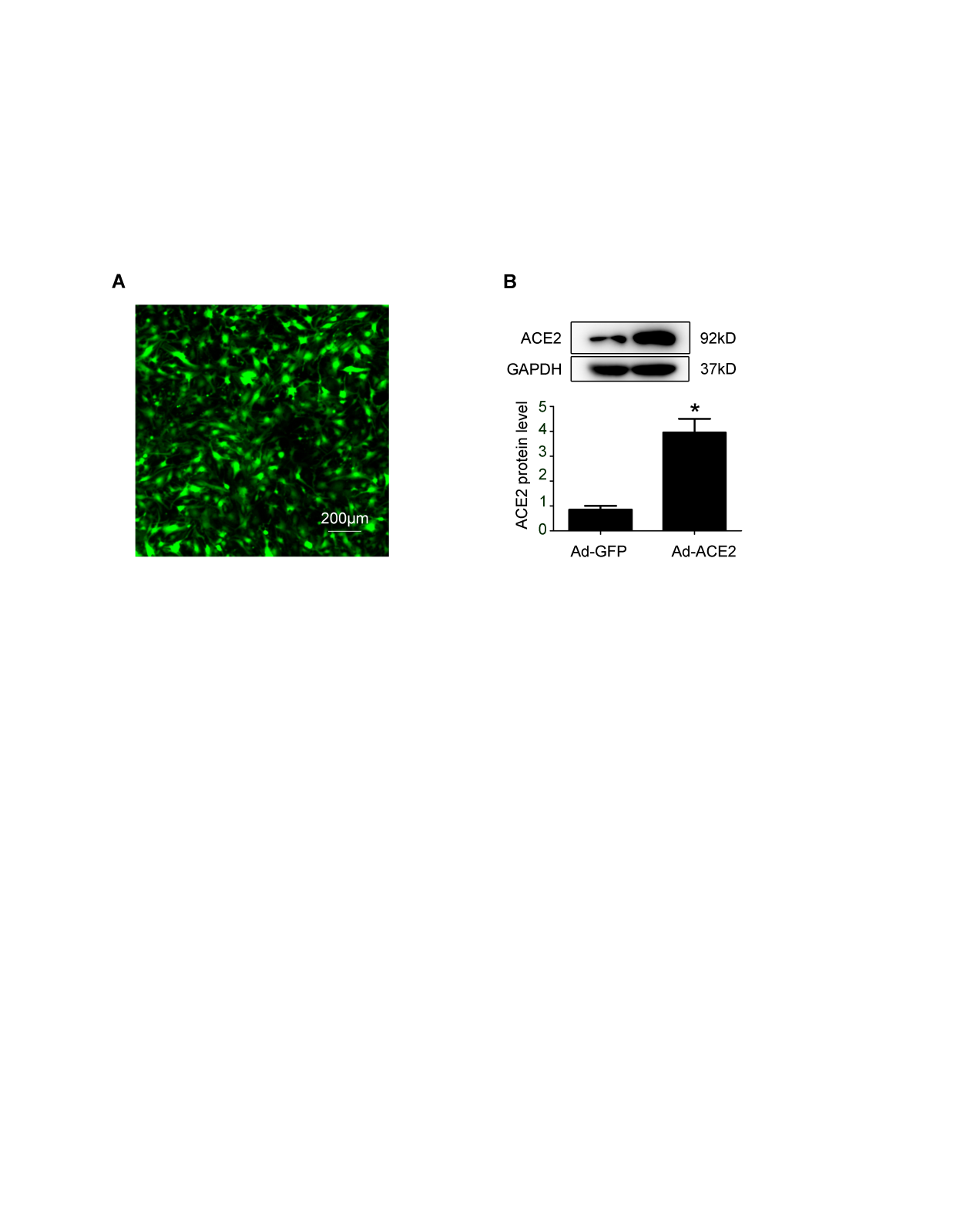


**Supplementary Figure 2.** ACE2 overexpression by adenovirus infection. Fluorescence images and Western blot analysis of ACE2 expression in HASMCs transfected with ACE2 adenovirus infection. Values are expressed as the means ± SEM. **P*＜0.05 versus Ad-GFP group (n=5).


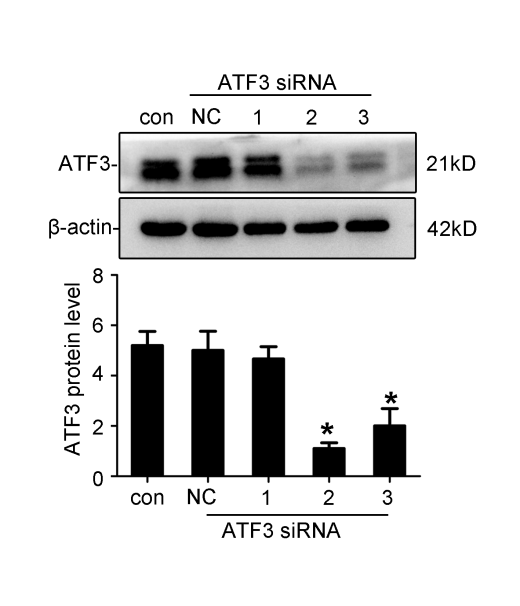


**Supplementary Figure 3.**  Western blot analysis of ATF3 expression in HASMCs transfected with ATF3 siRNA, demonstrating the efficiency of ATF3 knockdown. Values are expressed as the means ± SEM. * P<0.05 versus con or NC group (n=4).


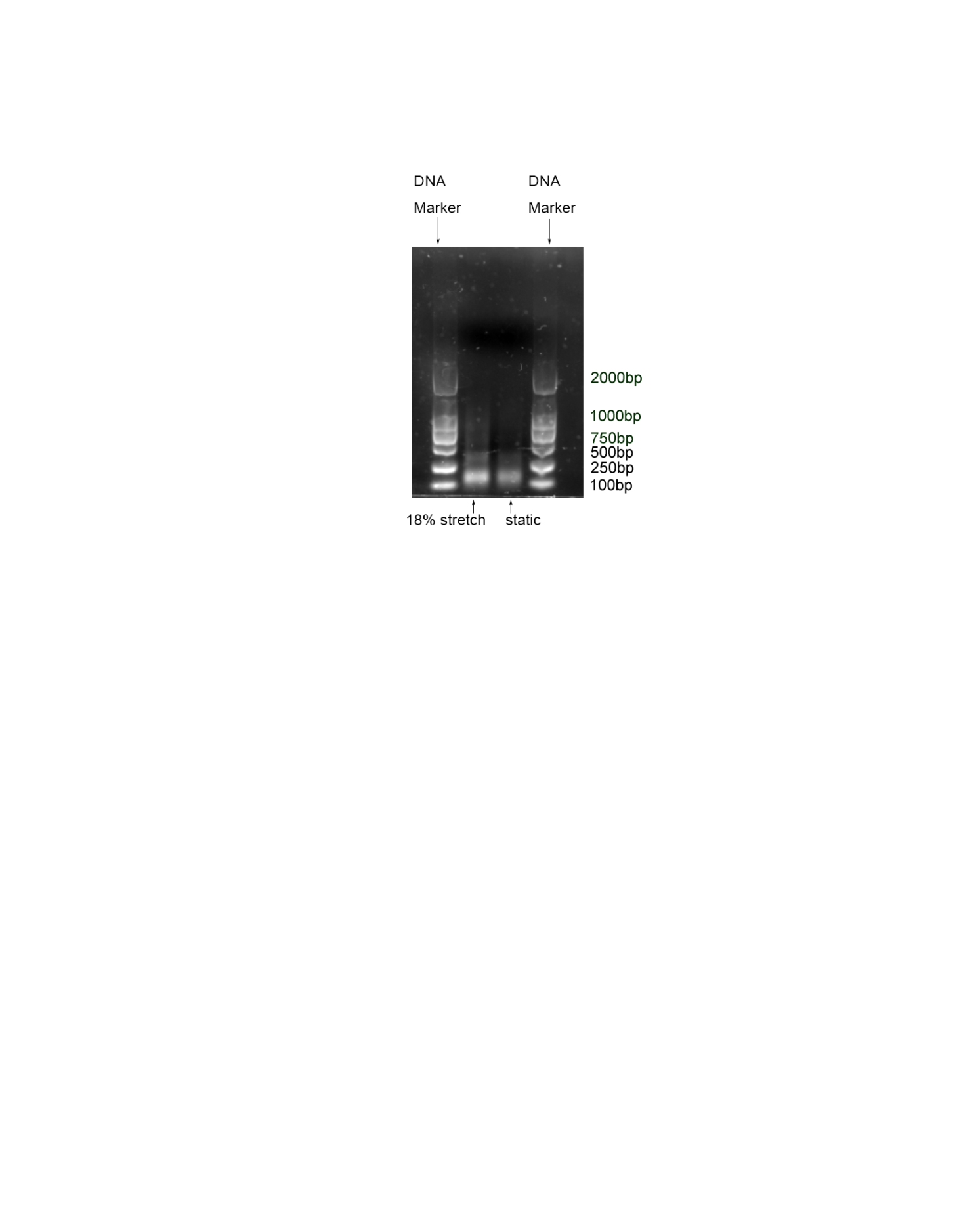


**Supplementary Figure 4.**  Human aortic smooth muscle cells were formaldehyde-crosslinked and chromatin was prepared and digested to fragments.


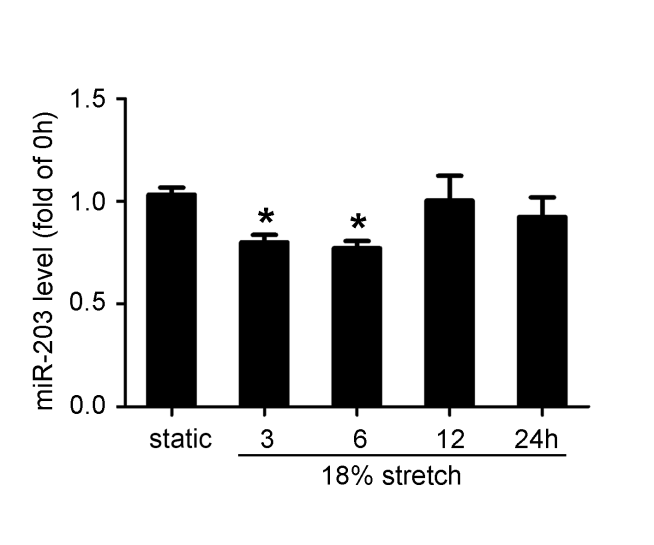


**Supplementary Figure 5.**  RT-qPCR analysis of the levels of miR-203 under 18% mechanical stretch for indicated time. sValues are expressed as the means ± SEM. * P<0.05 versus static group (n=4).


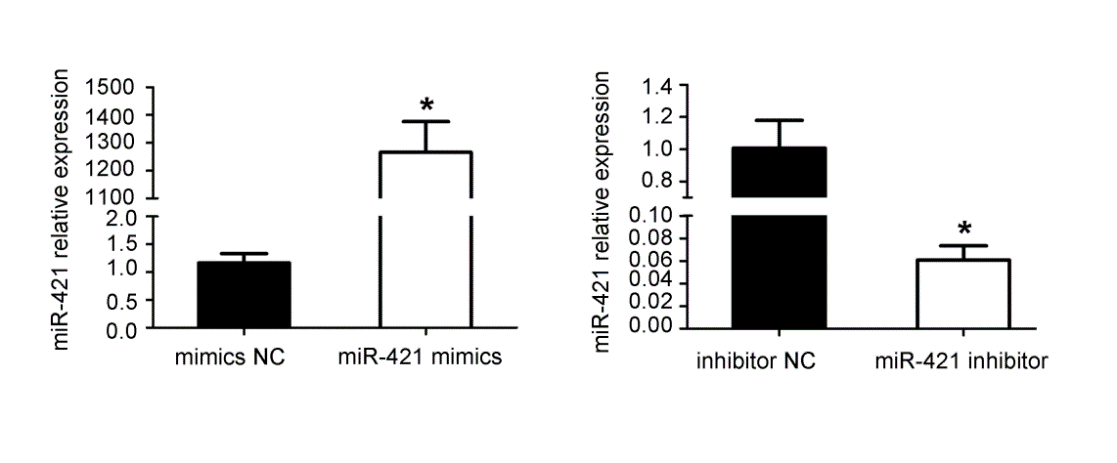


**Supplementary Figure 6.** RT-qPCR analysis of miR-421 expression in VSMCs transfected with miR-421 mimics and inhibitor after 24 h. Values are expressed as the means ± SEM. * P<0.05 versus mimics NC or inhibitor NC group (n=4).


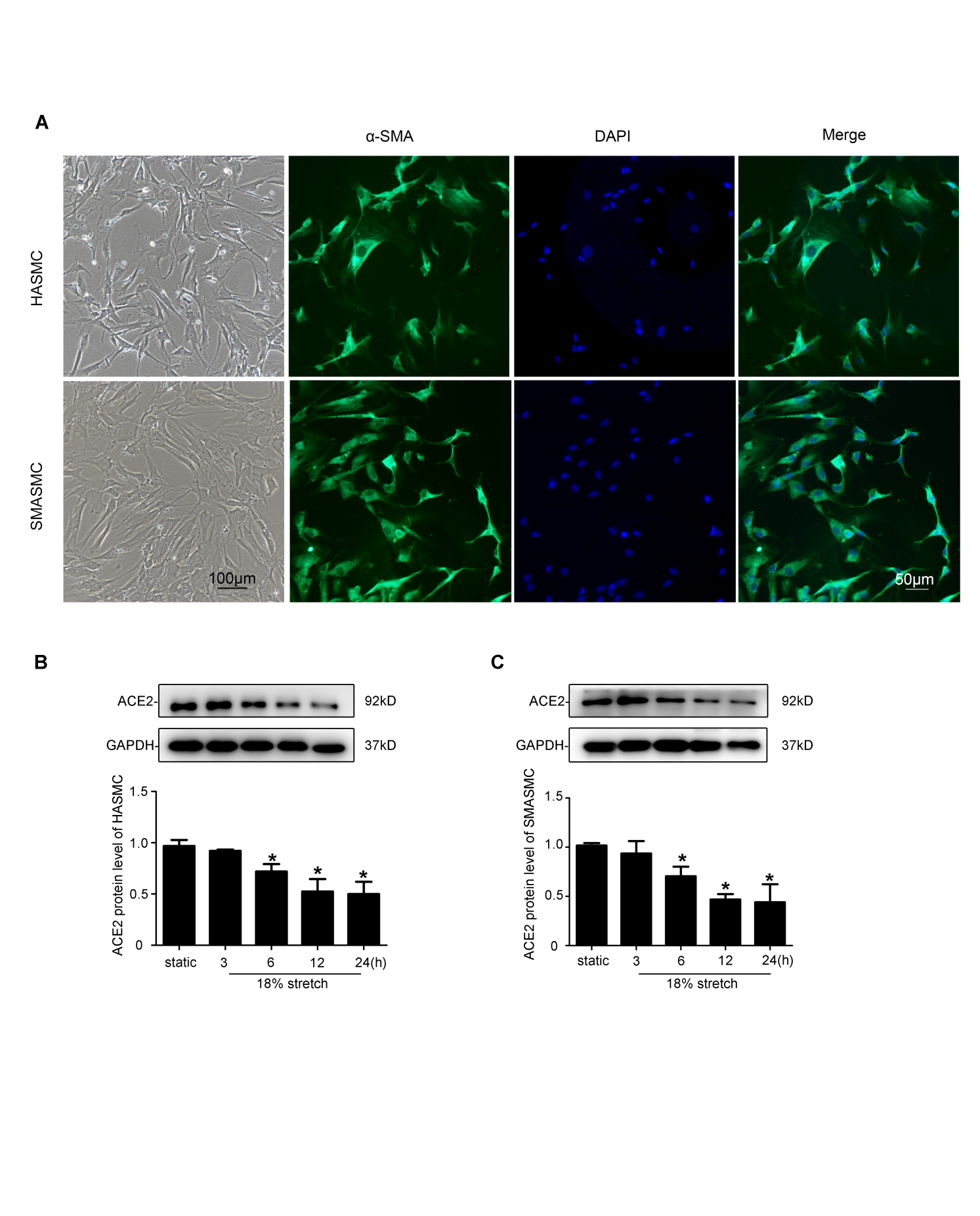


**Supplementary Figure 7.** (A) Representative photomicrographs of morphology and α-SMA expression of HASMCs and SMASMCs. (B and C) Western blot analysis of ACE2 protein expression in HASMCs and SMASMCs under mechanical stretch for indicated time. Values are expressed as the means ± SEM. * P<0.05 versus static group (n=4).
